# Indicators of Early and Late Processing Reveal the Importance of Within-Trial-Time for Theories of Associative Learning

**DOI:** 10.1371/journal.pone.0066291

**Published:** 2013-06-24

**Authors:** Harald Lachnit, Anna Thorwart, Holger Schultheis, Anja Lotz, Stephan Koenig, Metin Uengoer

**Affiliations:** 1 Fachbereich Psychologie, Philipps-Universität Marburg, Marburg, Germany; 2 Fachbereich Informatik, Universität Bremen, Bremen, Germany; University College London, United Kingdom

## Abstract

In four human learning experiments (Pavlovian skin conductance, causal learning, speeded classification task), we evaluated several associative learning theories that assume either an elemental (modified unique cue model and Harris’ model) or a configural (Pearce’s configural theory and an extension of it) form of stimulus processing. The experiments used two modified patterning problems (A/B/C+, AB/BC/AC+ vs. ABC-; A+, BC+ vs. ABC-). Pearce’s configural theory successfully predicted all of our data reflecting early stimulus processing, while the predictions of the elemental theories were in accord with all of our data reflecting later stages of stimulus processing. Our results suggest that the form of stimulus representation depends on the amount of time available for stimulus processing. Our findings highlight the necessity to investigate stimulus processing during conditioning on a finer time scale than usually done in contemporary research.

## Introduction

In any situation in which a stimulus has useful predictive value, this stimulus is embedded within an array of other stimuli, at the very least those that comprise the learning context. Hence, a basic question, with which associative learning theorists have struggled for many years, is whether learning attaches independently to the elements that constitute the entire sensory array or whether it attaches instead to the array as a whole. Theories that adopt the former view, so-called elemental theories, assume that responding to an array composed of many elements is a direct function of the values attached to the elements themselves, with the whole array having no separate value over and above that of its constituent parts. In contrast, theories that posit that the basic units of learning are entire arrays (‘configural’ theories) presume that responding is driven by knowledge about the whole array, independently of what its parts may signify. In the present experiments we will mainly focus on four currently discussed approaches, two elemental models and two configural models. As representative of elemental models we took a modified unique cue approach suggested by Redhead and Pearce [1] and the model of Harris [2], whereas representatives of configural models are the configural theory of Pearce [3] and an extended version of it suggested by Kinder and Lachnit [4] (for further detail see Lachnit, Schultheis, König, Üngör, and Melchers [5]).

Most of the empirical evaluation of elemental and configural theories so far was done either with eyeblink conditioning in rabbits using multimodal stimuli [6]–[8] or with autoshaping in pigeons using visual stimuli [1], [9], [10]. Several researchers have claimed that the kind of experimental stimuli used may strongly influence whether these stimuli are processed configurally or elementally [7], [11]–[13] (for a more extensive review see Melchers, Shanks, & Lachnit [14] and Shanks, Lachnit, & Melchers [15]). In the domain of animal learning, perhaps the most obvious aspect in this regard is the use of uni- versus multimodal stimuli. With multimodal compounds, considerable evidence in favor of elemental theories has been found, whereas studies using unimodal compounds have favored Pearce’s position.

In contrast to these studies, however, results from several recent investigations have questioned this conclusion. Kinder and Lachnit [4], for example, used two of the discrimination problems, which were originally introduced by Redhead and Pearce [1], in eyeblink conditioning experiments in humans with unimodal (visual) compounds. They compared the predictions of the Pearce model with those of the unique cue model and found support for neither the unique cue hypothesis nor the Pearce model. Only a post hoc extension of Pearce’s configural theory was able to account for these data. Similarly, Deisig, Lachnit, Sandoz, Lober, and Giurfa [16] used two discrimination problems suggested by Redhead and Pearce [1] to compare elemental and configural approaches. They conditioned the proboscis extension reflex of honeybees with unimodal (olfactory) compounds. Their data provided some support for an elemental approach. Glautier [17] also used only visual stimuli in a causal learning scenario where human participants had to learn which of various planes caused high levels of pollution. The data was again consistent with an elemental model but contradicted the predictions of the Pearce model. Last but not least, Lachnit et al. [5] compared various elemental theories with the Pearce model and its extension in two causal learning experiments with human participants using visual stimuli (pictures of fruits) in modified patterning problems initially suggested by Redhead and Pearce. Only two elemental models, the Harris model and the modified unique cue approach, successfully accounted for all of the data.

This diverse body of evidence indicates that the distinction of unimodal and multimodal stimuli might not be as important as assumed in the literature. Melchers, Shanks, and Lachnit [14] argued that there might well be a flexibility in the processing of stimuli, speaking against an either/or decision in the elemental/configural debate. They specified some potential mediating factors including task demands. A further factor that was not taken into account, however, is the potential impact of processing time. Lamberts [18], for example, presented a model for categorization describing the within trial time course. He claimed that perceived similarity varies with processing time, depending on the time course of inclusion of different stimulus dimensions into the similarity computation. In a similar vein, Lockhead [19] suggested that any object is initially perceived at some locus in representational space. This means that early in processing each object is processed holistically, and analytic (or elemental) processing occurs later, but only if the task demands such processing. If Lockhead’s model is valid, rapid responding should be associated with more holistic responding, whereas slow responding could be associated with more analytic responding. Ward [20] examined this relation and found evidence for a holistic-to-analytic processing sequence in visual perception.

Before associations can be built up, stimuli have to be represented, and these representations might change within trials. Perhaps, initial processing only yields a crude “holistic” first impression, whereas late processing reflects a more refined analysis using dimensional information.

On the basis of these theoretical accounts, some theoretical reframing of the preliminary findings should take place. For instance, most of the data in support of Pearce’s configural theory was derived from autoshaping experiments. There are two sources of autoshaped key pecking: conditioned responding and orienting [21]–[24]. Given the latter response component, it is reasonable to consider autoshaped key pecking as a response indicating early stimulus processing. However, in eyeblink conditioning experiments, the conditioned eyeblink response is usually defined as a response occurring shortly before the US. The parametrization excludes orienting responses to CS onset. Therefore, eyeblink responses in these experiments can be considered to be a rather pure measure of associative strength as well as a response indicating later stages of stimulus processing. Similarly, judgments and predictions recorded in causal learning experiments in which the participants worked self-paced can also be considered as responses indicating late stimulus processing. Thus, one procedural difference between the previous experiments, which might be responsible for the diverging results, might be that the various responses under investigation reflected different stages of stimulus processing.

Following Lachnit et al. [5], the present experiments were designed to compare the predictions of two elemental models with those of two configural models within a series of four experiments with humans. In two of these experiments (Experiments 1 and 2), we conditioned the skin conductance response (SCR) using a parametrization in which early and late responses within a trial can be distinguished (for details, see Experiment 1). As a further indicator of late stimulus processing, we also conducted one causal learning experiment in which the participants worked self-paced (Experiment 3). As a further indicator of early stimulus processing, we finally conducted a reaction time experiment in which we limited the amount of time available for categorization (Experiment 4).

All present experiments used the same kind of unimodal (visual) stimuli, which were composed of small colored rectangles (see also Redhead and Pearce [1]). The two discrimination problems used in our evaluation were also borrowed from Redhead and Pearce [1]. All discrimination problems used single stimuli, two-stimuli compounds, and triple compounds. One of these problems (A/B/C+, AB/AC/BC+ versus ABC-) was the same as used by Kinder and Lachnit [4] and by Lachnit et al. [5]. The second discrimination (A+, BC+ versus ABC-) was already used by Lachnit et al., too. These two discrimination problems were chosen because simulations of the various elemental and configural models generated by computer simulations (see Appendix S1) using the simulation tools of Schultheis, Thorwart, and Lachnit [25], [26] and Thorwart, Schultheis, König, and Lachnit [27] showed that focusing on the rank order of pre-asymptotic responding will in principal allow us to decide between the theories under consideration.

## Experiment 1: Pavlovian SCR Conditioning (A/B/C+, AB/BC/AC+ versus ABC-)

In SCR conditioning two distinct responses can be distinguished with an ISI of 8000 ms, first interval responses (FIR) and second interval responses (SIR). The FIR, measured as a response to CS onset, is known to be supplied by two different processes. On the one hand, it is a conditioned response, on the other hand, it is an orienting response [28]–[30]. The SIR, as a response antedating the US (like for example the eyeblink response) is considered to be a rather pure measure of associative strength. Under a different perspective, however, FIR could be looked at as indicating early stimulus processing and SIR as late stimulus processing.

In Experiment 1 we therefore used SCR conditioning to evaluate the rivaling elemental and configural theories. Stimuli and design (discrimination problem A/B/C+, AB/AC/BC+ versus ABC-) employed for Experiment 1 had been suggested by Redhead and Pearce [1] and were also used in Experiment 2 of Kinder and Lachnit [4]. In contrast to Redhead and Pearce, however, we used human participants instead of animals, and contrary to Kinder and Lachnit we used SCR conditioning instead of eyeblink conditioning. Furthermore, the design was the same as the negative patterning problem of Experiment 1 of Lachnit et al. [5]. Contrary to this experiment, in the present Experiment 1 the extended negative patterning discrimination problem was not trained interleaved with extended positive patterning.

Pre-asymptotically, both elemental models predict stronger responding to two-stimuli compounds than to single stimuli. Both configural theories predict stronger responding to single stimuli than to two-stimuli compounds. However, when increasing the discrimination parameter in the extended configural theory, this difference diminishes across trials.

With human causal learning, Lachnit et al. [5] found the empirical results to be in full agreement with the predictions of both elemental models. Kinder and Lachnit [4] using human eyeblink conditioning, however, found pre-asymptotically no difference in responding between the reinforced stimuli. One reason for these divergent results may have been that with causal learning the number of reinforced trials blocked together was much smaller (1 trial blocks) than with eyeblink conditioning (blocks of 12 trials). Using SCR conditioning instead of eyeblink conditioning allows for a substantial decrease in the number of trials per block (3 trials in case of the present experiment), and by this for a better possibility to detect differences in responding that occur early in training.

### Methods

#### Participants

Twenty-four students of the Philipps-Universität Marburg took part in the experiment. They either received course credit or were paid for participation. The participants were informed that the purpose of the experiment was to measure their physiological responses to various visual stimuli presented on a screen and occasional electric shocks. They were asked to relax, to avoid unnecessary movements and heavy breathing, and to pay close attention to the stimuli. The data of eight participants had to be excluded because they showed habituation of the unconditioned response (UR) in course of the experiment (more than 20% of the reinforced trials without UR). The age of the remaining 16 participants (12 females and 4 males) varied between 19 and 64 years with a mean age of 24.4 years. Participants gave informed written consent to participate in the experiment. The experimental procedure was approved by the ethics committee of the Psychology Department of the Philipps-Universität Marburg.

#### Stimuli and procedure

As already mentioned, the CSs were composed of small colored rectangles (5×7 mm) which were presented on a black background. Between trials the screen only consisted of the black background. From trial to trial the rectangles were randomly located in a matrix that could accommodate a maximum of 42 rectangles horizontally and 32 rectangles vertically. On A, B, and C trials 100 rectangles of one color were presented. On AB, AC, and BC trials 50 rectangles of one color and 50 rectangles of another color appeared on the screen. On ABC trials 33 rectangle of each color appeared on the screen. For half of the participants, the colors were red, yellow and green, for the other half magenta, brown and blue. Because an analysis of variance (ANOVA) revealed no differences between these two, sets all analyses reported were collapsed across these two subgroups. The assignment of the colors to the elemental stimuli was balanced across participants. The CSs lasted for 8 s. A dc electric shock served as US. The shock was delivered via silver-silver chloride electrodes to the volar surface of the participant’s left arm from an isolated transformer-condenser shock generator [31]. The intensity of the shock was adjusted together with the participant so that it was “definitely unpleasant but not really painful”. Shock duration was about 20 ms. On reinforced trials, the shock was delivered simultaneously with CS-offset. The interval between consecutive trials (CS-onset to CS-onset) was 24 s on average, plus or minus 2 s. The administration and timing of the stimuli were computer-controlled. Palmar skin conductance was picked up from the thenar and hypothenar eminences of the participants’ right hand by silver-silver chloride electrodes, 0.8 cm in diameter, which were filled with an isotonic electrolytic medium. Prior to attaching the electrodes the skin was cleaned. Skin conductance was processed with a constant voltage bridge as described by Lykken and Venables [32] and sampled by a computer at 20 Hz.

The experiment took place in a sound-attenuated and well-illuminated room. Participants were seated 135 cm in front of the computer display that was used for CS presentation. Each participant received 36 training trials, on half of which a US was presented. Each reinforced stimulus (A, B, C, AB, AC, and BC) was presented three times. The ABC- stimulus was presented 18 times. Reinforced and nonreinforced trials were presented in a pseudo-randomized order with a maximum of three reinforced or nonreinforced CSs occurring consecutively.

#### Dependent variable

The first interval response, FIR, is defined as the maximum conductance change of responses beginning during the interval 1–4 s after CS onset (i.e., the difference between the skin conductance level at the peak of the response and the value just at its onset). The second interval response, SIR, is defined as the maximum conductance change beginning during the interval 4–9 s after CS onset. Conductance changes were converted to logarithmic values (after adding 1), as recommended by Venables and Christie [33], and then multiplied by 1000.

An ANOVA revealed no significant difference between the three single stimuli and the three two-stimuli compounds. Hence, the data of A, B and C as well as of AB, BC and AC were combined. Trials were averaged in blocks of 3 trials, resulting in 3 A/B/C+ and 3 AB/BC/AC+ blocks. Accordingly, the three ABC- trial blocks consisted of 6 trials each.

### Results and Discussion

The.05 level of significance was used for all statistical tests for this and the following experiments. Stated probability levels of all ANOVAs are based on the Huynh and Feldt [34] adjustment where appropriate.

First, in order to evaluate whether or not responding correctly mirrored the contingencies, we collapsed the data across the reinforced single stimuli and two-stimuli compounds and compared these by means of a 2×3 (Contingency×Block) ANOVA with the nonreinforced triple compound. Second, we conducted a 2×3 (Stimulus×Block) ANOVA in order to compare responding to the single stimuli and the two-stimuli compound. In all cases we will mainly focus on effects related to learning (main effect of stimulus or contingency, interaction of these effects with block). Main effects of block will only be reported in relation to the question whether or not there is pre-asymptotic or asymptotic behavior.

**Figure 1:**
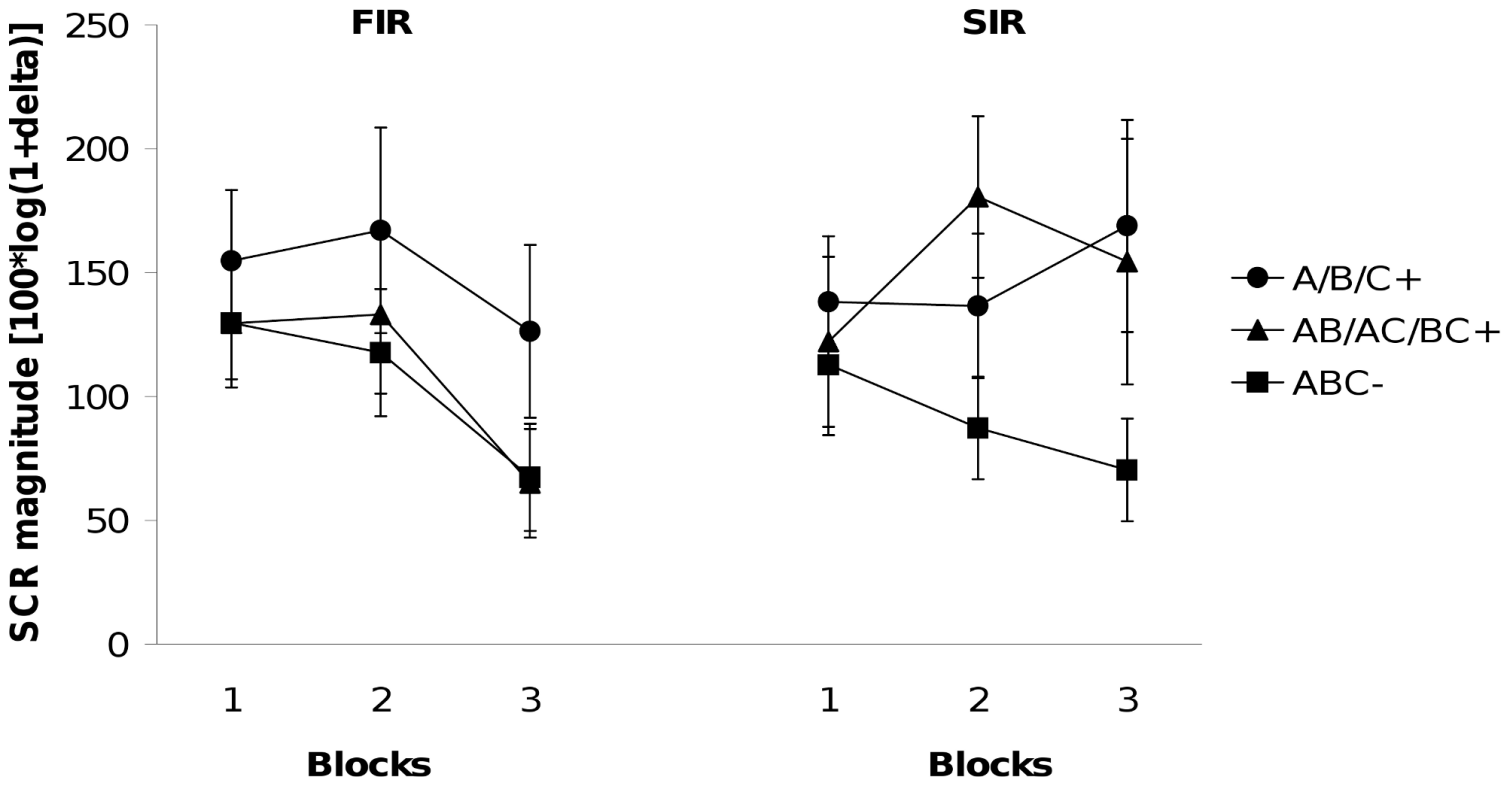
Experiment 1. Mean magnitudes of skin conductance responses during acquisition. (Left-hand panel shows first interval responses [FIR]; right-hand panel shows second interval responses [SIR]). Responses are shown in blocks of three trials for reinforced stimuli and in blocks of six trials for ABC-.

The left hand panel of Figure 1 shows the results for the FIR. The 2×3 (Contingency×Block) ANOVA revealed a significant main effect of contingency, *F* (1, 15) = 6.98, *p*<.02. Inspection of Figure 1 shows that averaged responding to the stimuli signaling shock (A/B/C+, AB/AC/BC+) exceeded responding to those presented without shock (ABC-). The Contingency×Block interaction was not significant, *F* <1, indicating that the amount of response differentiation did not change across blocks. The 2×3 (Stimulus×Block) ANOVA showed that responding to A/B/C+ was significantly stronger than to AB/AC/BC+, *F* (1, 15) = 6.55, *p*<.03. This difference did also not change significantly throughout training (Stimulus×Block interaction: *F* <1).

In case of SIR (right hand panel of Figure 1), differential conditioning was successful, too. The 2×3 (Contingency×Block) ANOVA revealed a significant main effect of contingency, *F* (1, 15) = 10.32, p<.007, while the interaction was not significant, *F* (2, 30) = 2.41, p>.12. A 2×3 (Stimulus×Block) ANOVA of the single stimuli and the two-stimuli compounds showed that responding to A/B/C+ did not differ at all from responding to AB/AC/BC+, *F* <1, and the Stimulus×Block interaction was also not significant, *F*(2, 30)* = *1.43, p>.25. The main effect of block, *F* <1, was not significant either.

To compare FIR and SIR, we conducted an Interval (FIR vs. SIR)×Stimulus (single cues vs. two-stimuli compounds)×Block (1–3) ANOVA. The main effect of interval fell short of significance, *F*(1, 15)* = *3.66, *p*>.07, as well as the Interval×Block interaction, *F*(2, 30)* = *3.19, *p*>.07. The remaining main effects and interactions were not significant, all *F*s <2.44, all *p*s >.13.

Experiment 1 yielded surprising results in several respects. Although FIR and SIR were measured within one experiment and within subjects, we found diverging results across the two intervals. For the FIR, we observed stronger responding to single stimuli than to two-stimuli compounds. This finding only conforms to the predictions of the configural theory, while the elemental models and the extended configural theory clearly failed to predict the observed rank order of responding.

SIR conditioning, however, yielded quite different results. For the second interval, we observed no differences in responding to single stimuli and two-stimuli compounds, and responding to both did not increase across blocks. This finding converged more with the eyeblink data of Kinder and Lachnit [4] than with the causal learning data of Lachnit et al. [5], although the number of trials per block had substantially been decreased in order to ensure the possibility to detect differences between the reinforced single stimuli and the two-stimuli compounds. As our SIR data are indicative of asymptotic learning, both elemental theories and the extended configural theory correctly predict the results observed. If, on the other hand, the data represent pre-asymptotic learning (the more implausible case), only the extended configural theory is able to correctly predict the data. Regardless of the question whether SIR learning was pre-asymptotic or asymptotic, the configural theory is unable to deal with our findings from the second interval.

Thus, if we consider the FIR data as measures of early stimulus processing and the SIR data as measures of late processing within a trial, the results suggest that early in a trial configural processing is dominant, whereas later elemental processing prevails (elemental theories) or generalization decreases (extended configural theory). This view conforms to Lamberts’ model of categorization [18].

The results of Experiment 1 indicated a difference in FIR and SIR. However, our conclusions drawn from the present experiment are not entirely satisfactorily supported by the statistical analyses as the direct comparison between FIR and SIR results revealed no significant difference. One reason for this might be the great variability of the SCR data across participants, which might be caused by the difficulty of the present discrimination problem. Therefore, in Experiment 2, which was intended to confirm the reliability and generality of the results from Experiment 1, we used a simpler discrimination problem in order to enhance the probability of finding significant differences between FIR and SIR.

In Experiment 2, we employed the design of Experiment 2 of Lachnit et al. [5], namely the A+/BC+ versus ABC- design suggested by Redhead and Pearce [1]. With human causal learning, Lachnit et al. [5] found that pre-asymptotically responding to the single stimulus and to the two-stimuli compound did not differ, exactly what the Harris model with no or a very low fraction of common elements (.1), the modified unique cue model, and the extended configural theory predict. Configural theory, on the other hand, pre-asymptotically predicts higher responding to the single stimulus than to the two-stimuli compound.

## Experiment 2: Pavlovian SCR-Conditioning (A+, BC+ versus ABC-)

Compared to Experiment 1, in which seven different CSs had to be discriminated, Experiment 2 with only three different CSs should be much easier. We therefore shortened the training from 36 to 24 trials, and instead of computing blocks of 3 and 6 trials, we now looked at blocks of 2 and 4 trials, respectively.

In light of the results of Experiment 1, we should again find diverging results in early (FIR) and late (SIR) processing, with early processing results being best predicted by Pearce’s configural theory, while late processing results are in support of the three other theories.

### Methods

The methods were the same as in Experiment 1. Therefore only changes will be described here.

#### Participants

Twenty-five students of the Philipps-Universität Marburg took part in the experiment. None of them had participated in one of the other experiments. The data of one participant had to be excluded due to habituation of the UR. The age of the remaining 24 participants (22 females and 2 males) varied between 19 and 46 years with a mean age of 22.0 years. Participants gave informed written consent to participate in the experiment.

#### Stimuli and procedure

The same US and some of the CSs were used as in Experiment 1, but the colors were red, blue and green for all participants. Each participant received 24 training trials, on half of which a US was presented. There were 6 A+, 6 BC+ and 12 ABC- trials. The first two trials were restricted to four sequences (A+/ABC-, BC+/ABC-, ABC−/A+, ABC−/BC+) and balanced over the participants.

#### Dependent variable

FIR and SIR were defined and measured as in Experiment 1. Trials were averaged in blocks of 2 trials for A+ and BC+, and blocks of 4 trials for ABC-.

### Results and Discussion

**Figure 2:**
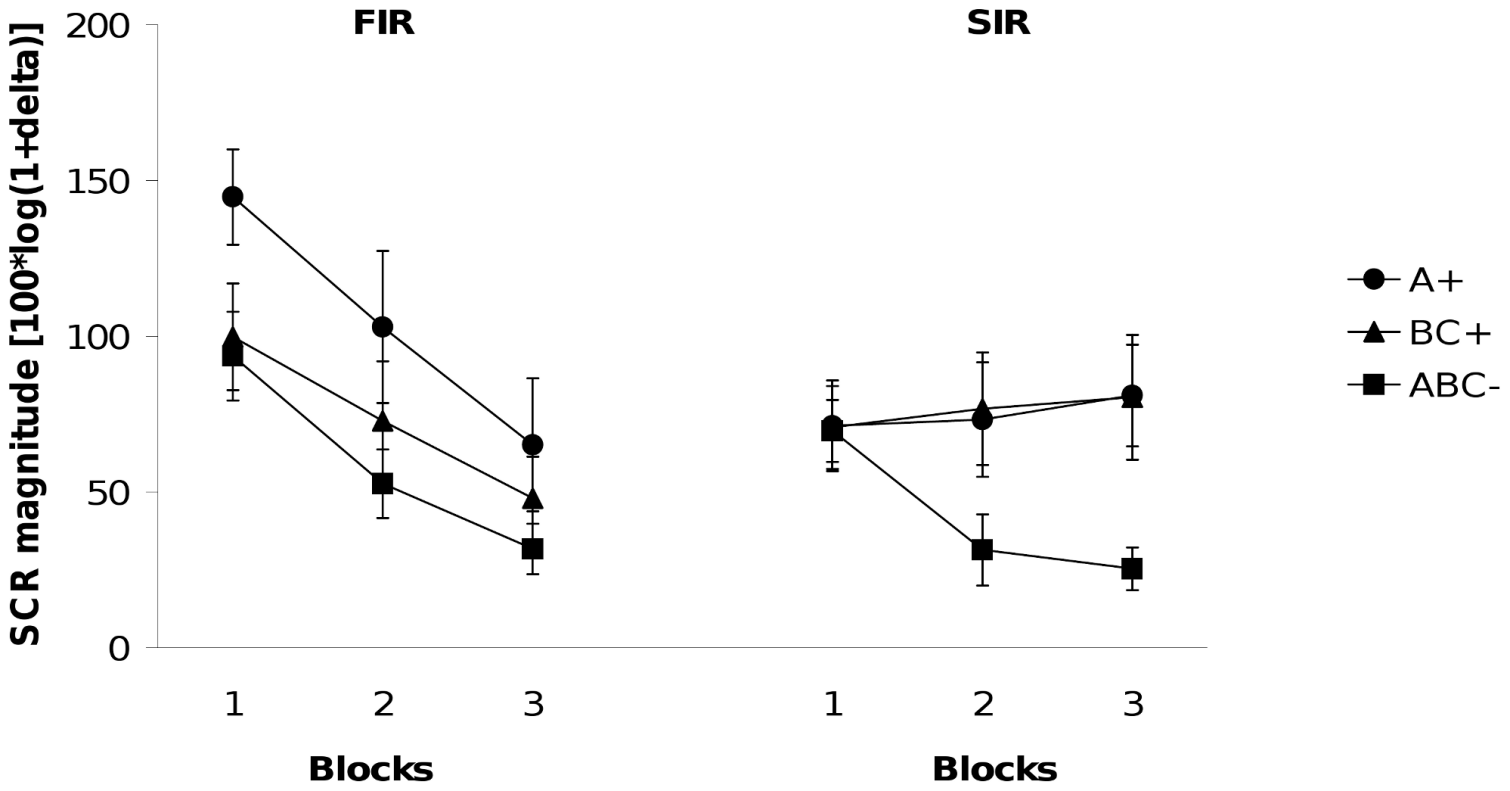
Experiment 2. Mean magnitudes of skin conductance responses during acquisition. (Left-hand panel shows first interval responses [FIR]; right-hand panel shows second interval responses [SIR]). Responses are shown in blocks of two trials for reinforced stimuli, A+ and BC+, and in blocks of four trials for ABC-.

The left hand panel of Figure 2 shows the results of the FIR. The 2×3 (Contingency×Block) ANOVA revealed a significant main effect of contingency, *F* (1, 23) = 9.36, *p*<.007, showing that responding to A+ and BC+ exceeded responding to ABC-. The Contingency×Block interaction was not significant (*F* <1). The 2×3 (Stimulus×Block) ANOVA revealed a main effect of stimulus showing that responding to A+ was significantly stronger than to BC+, *F* (1, 23) = 6.02, *p*<.03. This difference did not change significantly throughout training (Stimulus×Block interaction: *F* <1).

In case of SIR (right hand panel of Figure 2) the 2×3 (Contingency×Block) ANOVA revealed a significant main effect of contingency, *F* (1, 23) = 8.45, p<.009, and a significant Contingency×Block interaction, *F* (2, 46) = 5.30, p<.01. The 2×3 (Stimulus×Block) ANOVA showed that responding to A+ did not differ at all from responding to BC+ (*F* <1). The Stimulus×Block interaction and the main effect of block were also not significant, both *F*s <1.

To compare FIR and SIR, we conducted an Interval (FIR vs. SIR) × Stimulus (single cues vs. two-stimuli compounds)×Block (1–3) ANOVA. There was no main effect of stimulus, *F*(1, 23)* = *3.02, *p*>.09, and no Stimulus×Block interaction, *F* <1. However, the analyses revealed a significant Interval×Stimulus interaction, *F*(1, 23)* = *4.47, *p*<.045, showing that discriminative responding differed across FIR and SIR.

As in Experiment 1, the FIR showed stronger responding to A+ than to BC+. This conforms to the predictions of the configural theory. In SIR, responding to A+ and to BC+ did not differ at all. As our SIR data indicated pre-asymptotic learning, both elemental theories and the extended configural theory correctly predict the results observed, while the configural theory is unable to deal with our findings from the second interval.

## Experiment 3: Extended Negative Patterning in Causal Learning

As mentioned in the introduction, Lachnit et al. [5] investigated modified patterning problems using a causal learning procedure. In their Experiment 1, for instance, Lachnit et al. trained participants with an extended negative patterning problem (A/B/C+, AB/BC/AC+ vs. ABC-) similar to the one used in our present Experiment 1. However, in contrast to the present series of experiments, Lachnit et al. used different pictures of fruits as visual stimuli. In order to further tie together the causal learning studies of Lachnit et al. [5] and the present Pavlovian conditioning experiments, Experiment 3 was designed as a causal learning study, but using the colored rectangles instead of pictures of fruits.

In causal learning experiments, participants typically perform self-paced tasks. That is, the cues were shown until the participant makes a prediction. Thus, it is reasonable to consider responding in these experiments as indicating rather late stages of stimulus processing. If our analysis of the results of the previous experiments in terms of early and late stages of stimulus processing is correct, the results of Experiment 3 should be consistent with the predictions of the elemental theories or the extended configural theory, while the predictions of the configural theory should not be supported.

### Methods

#### Participants

Thirty six students of the Philipps-Universität Marburg (31 female, 5 male) with a median age of 21 (19–41) either participated to meet a course requirement or were paid for their participation. They were tested individually and required approximately 15 min to complete the experiment. Participants gave informed written consent to participate in the experiment. The experimental procedure was approved by the ethics committee of the Psychology Department of the Philipps-Universität Marburg.

#### Apparatus and procedure

The instructions were presented on a PC computer screen, and the participants gave their answers by using the mouse or the computer keyboard. As in the previous experiments, the stimuli were made up of colored rectangles (5 mm×7 mm) randomly located across trials in a matrix accommodating a maximum of 42 rectangles horizontally and 32 rectangles vertically. The rectangles were blue, brown, green, magenta, red, or yellow. The assignment of colors to stimuli was counterbalanced across participants. The area within the 42×32 matrix unoccupied by the colored rectangles appeared dark. Each of the single cues consisted of 100 rectangles of the same color, each of the two-stimuli compounds consisted of 50 rectangles of each color, and each of the three-stimuli compounds was composed of 33 rectangles of each color. The three different outcomes were “diarrhea” (O_1_), “abdominal pain” (O_2_), and “no symptoms” (-).

Participants were told to imagine that they were a physician who worked in a laboratory conducting bacteria analyses to discover which kinds of bacteria lead to medical disturbances. Participants were told that they would be shown several bacterial working stocks in successive order and that they must predict for each whether the person exposed to it would show medical disturbances or not. Participants were also told that they would receive corrective feedback after each prediction. They were asked to use this feedback to learn about the existing relationships and to make as many correct predictions as possible.

The learning stage consisted of six trials each of A→O_2_, B→O_2_, C→O_2_, AB→O_2_, AC→O_2_, BC→O_2_, and 24 ABC- trials (extended negative patterning), together with six trials each of D-, E-, F-, DE-, DF-, EF-, and 24 DEF→O_1_ trials (extended positive patterning). The learning stage was divided in six blocks. Each block consisted of one presentation of each of the single cues and of each of the two-stimuli compounds as well as of four presentations of each of the triple compounds. Thus, participants were shown 120 trials during the entire experiment. The order of presentation within each block was determined randomly for each block and each participant.

On each learning trial, the 42×32 matrix was presented at the centre of the screen. Participants gave their prediction whether or not they expected that a symptom would occur by clicking on one of three answer buttons which were labeled *No symptom*, *Abdominal pain* and *Diarrhea*. After making a prediction, a feedback window told participants whether the person actually showed a symptom or not. Participants had to confirm that they had read the feedback by clicking on an “OK”-button. Then the next trial started.

### Results and Discussion

**Figure 3:**
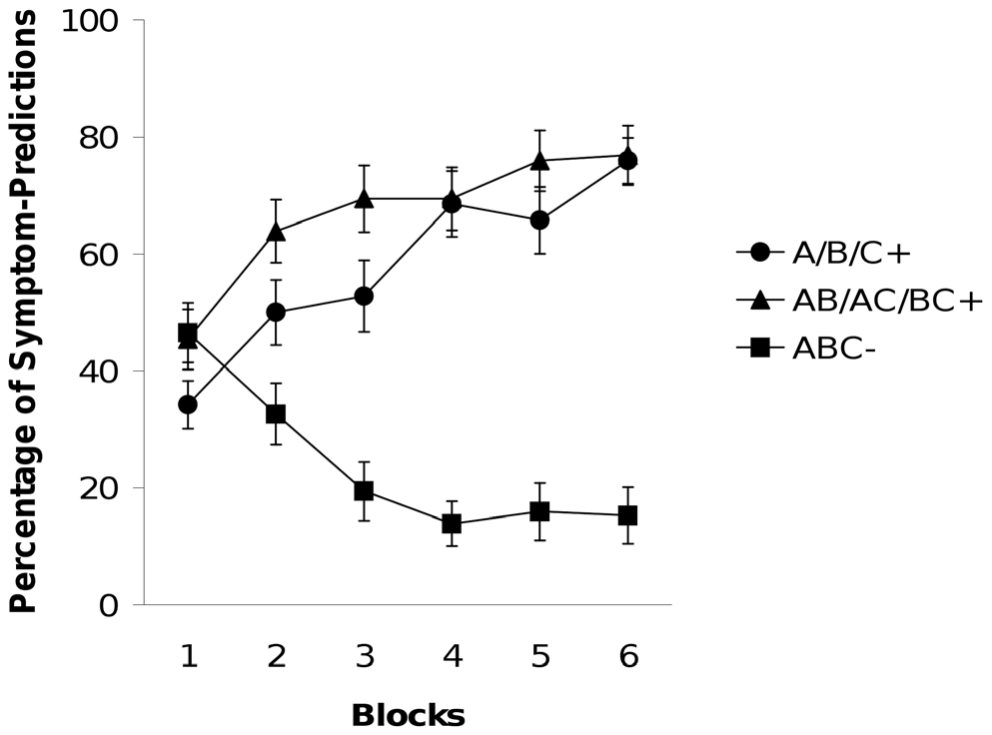
Experiment 3. Percentage of trials in each block in which participants predicted the relevant medical symptom in extended negative patterning problems. Blocks consisted of one presentation of single cues and one of two-stimuli compounds and four presentations of triple compounds.

Figure 3 shows the percentage of trials for which participants predicted the relevant symptom for the negative patterning problem. We neglected the positive patterning data, because the predictions of elemental and configural theories mainly differ with respect to negative patterning.

In a first step we checked for successful discrimination learning with a 2×6 (Contingency×Block) ANOVA. We found a significant main effect of contingency, *F* (1, 35) = 49.72, *p*<.001, and a significant Contingency×Block interaction, *F* (5, 175) = 27.77, *p*<.001.

Next, we compared responding to single cues and two-stimuli compounds. The 2×6 (Stimulus×Block) ANOVA revealed a significant main effect of stimulus, *F* (1, 35) = 4.33, *p*<.05, showing that responding to two-stimuli compounds was stronger than to single cues. There was no significant Stimulus×Block interaction, *F* (5, 175) = 1.24, *p*>.29.

In summary, for extended negative patterning the pattern of results nearly perfectly fitted to what had been observed with pictures of fruits as stimuli in Experiment 1 of Lachnit et al. [5]: higher responding to two-stimuli compounds than to single cues. This pattern of results is clearly inconsistent with the predictions of the configural theory. While the SIR data from each of our previous experiments did not allow us to differentiate between the extended configural theory and the elemental theories, the results from Experiment 3 are only anticipated by the elemental approaches.

## Experiment 4: Inducing Early Processing in a Categorization Task

The purpose of Experiment 4 was to investigate responding to single cues and two-stimuli compounds in extended negative patterning in a situation encouraging responses at an early stage of stimulus processing. Starting with the causal learning procedures of Experiment 3, we developed a categorization task in which we explicitly limited the amount of time available to categorize the stimuli. In order to achieve this goal for each participant, a time limit was determined on an individual base (see Procedure). Under time pressure, participants had to map single cues and two-stimuli compounds onto one key and triple compounds onto a second key.

If this procedure is suited to induce responses at an early stage of stimulus processing, we should observe a dramatic change in the pattern of results from the previous to the present experiment. While the results from Experiment 3 were in accord with elemental theories, in Experiment 4 we should observe a pattern of results consistent with configural approaches.

### Methods

#### Participants

Thirty six students of the Philipps-Universität Marburg (25 female, 11 male) with a median age of 22 (19–28) were tested individually and were paid for their participation. Participants gave informed written consent to participate in the experiment. The experimental procedure was approved by the ethics committee of the Psychology Department of the Philipps-Universität Marburg.

#### Apparatus and procedure

The instructions and stimuli were presented on a PC computer screen. Stimuli were the same as in Experiments 1–3. Participants made their responses by using a three-key response device. The response device was tagged by a white circle 3 cm in diameter. The three response keys were allocated on the left (L-key), on the right (R-key), and at the top (M-key) of the white circle. The assignment of the L-key and the R-key to the response-types R_1_ and R_3_ was counterbalanced across participants, whereas the M-key was constantly assigned to response-type R_2_.

Each participant initially worked on a training phase which was followed by a learning phase. For the training phase, participants were told that they would be shown the letters L, M, and R in successive order and that they would have to press the L-key, M-key, or R-key with the index finger of their preferred hand as quickly as possible whenever letter L, M, or R was presented, respectively. Each training trial started with the presentation of a fixation cross in the centre of the display for 1250 (±250) ms. With offset of the fixation cross, a letter was presented in the centre of the display for 1400 ms. The letter was replaced by a message telling the participants whether or not their reaction was correct and that they should respond as quickly as possible. This feedback appeared for 3 s. Thereafter the next training trial started. The training stage consisted of six trials each of L, M, and R and was divided into three blocks, each of which consisted of two presentations of each letter. The order of presentations was the same for all participants ({M, M, L, R, R, L}, {M, R, R, L, L, M}, {L, M, R, M, R, L}). For each participant, we averaged the reaction times across the correctly responded trials within the last two blocks as an estimate for the participant’s basic speed of responding.

For the learning phase, participants were told that they would be shown a number of colored squares on each of several trials. Participants were asked to press with the index finger of the preferred hand as quickly as possible the L-key, M-key, or R-key depending on the color or combinations of colors of the squares. Participants were also told that they would receive feedback after each trial whether the reaction was correct or wrong, and whether the reaction was fast enough or too slow. They were informed that on each trial they could gain or lose points depending on their reaction: a correct and fast enough reaction would result in gaining three points, whereas a wrong or a correct but too slow reaction would result in losing one point. Each learning trial started with the presentation of a fixation cross in the centre of the display for 1250 (±250) ms. With offset of the fixation cross, the 42×32 matrix was presented at the centre of the screen. The duration of the presentation of the matrix was individually pre-assigned for each participant (see below). The matrix was replaced by a message telling the participants whether or not their reaction was correct and whether or not they reacted fast enough or too slow. This feedback appeared for 5 s. Thereafter the next training trial started.

For each participant, the duration of the presentation of the matrix was determined by the respective mean reaction time calculated from the training phase plus 500 ms. The criterion for a reaction being fast enough or too slow was also individually pre-assigned for each participant (mean reaction time plus 220 ms).

The learning stage consisted of nine trials each of A→R_1_, B→R_1_, C→R_1_, AB→R_1_, AC→R_1_, BC→R_1_, and 36 ABC→R_2_ trials (extended negative patterning), together with nine trials each of D→R_2_, E→R_2_, F→R_2_, DE→R_2_, DF→R_2_, EF→R_2_, and 36 DEF→R_3_ trials (extended positive patterning). The learning stage was divided into nine blocks. Each block consisted of one presentation of each of the single cues and of each of the two-stimuli compounds as well as of four presentations of each of the triple compounds. Thus, participants were shown 180 trials during the entire learning phase. The order of presentation within each block was determined randomly for each block and each participant.

### Results and Discussion

**Figure 4:**
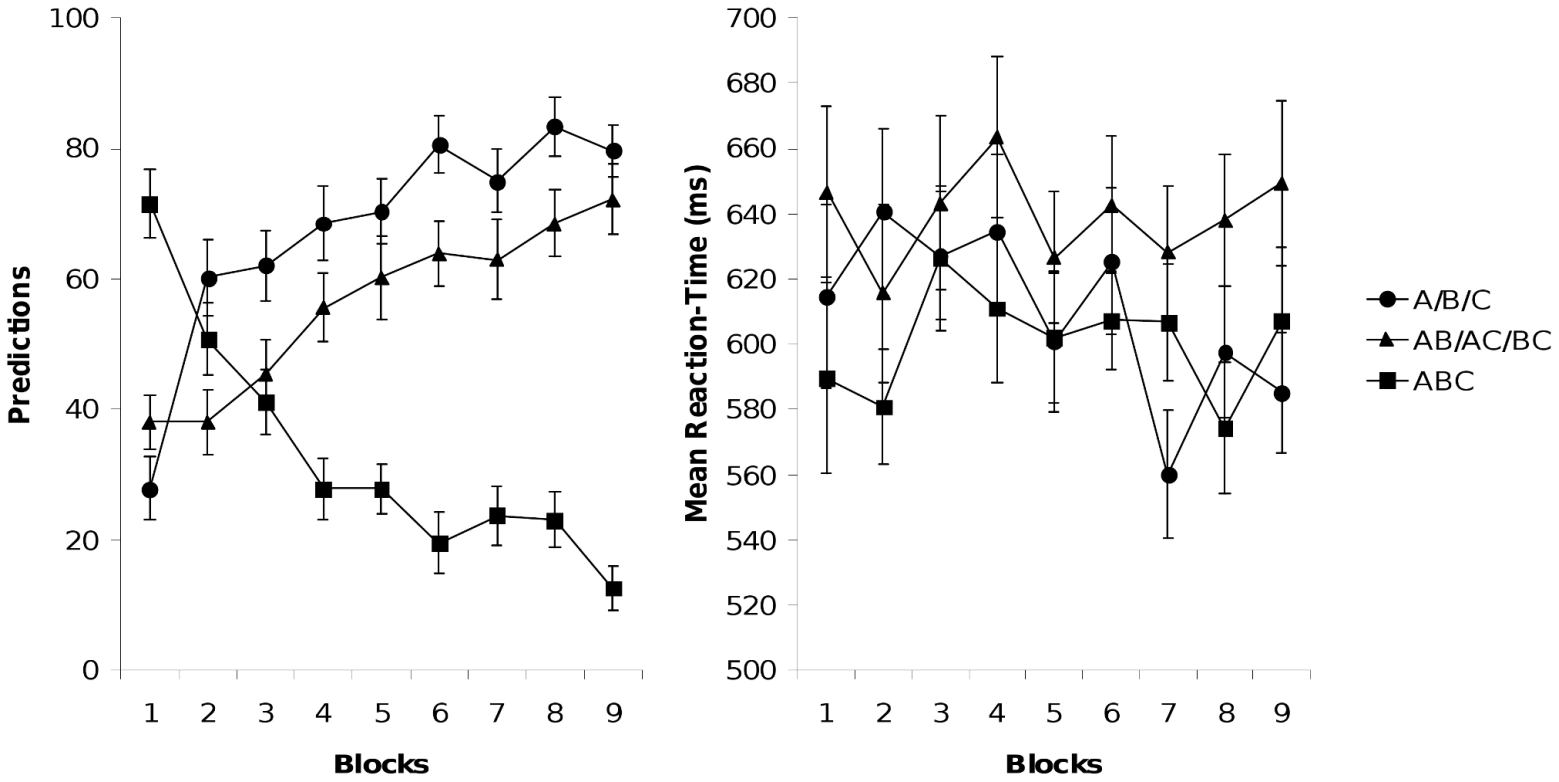
Experiment 4. Left-hand: Percentage of trials in each block in which participants made the relevant response to single cues, two-stimuli compounds, and three-stimuli compounds of the negative patterning problem (A/B/C→R_1_, AB/AC/BC→R_1_, and ABC→R_2_). Right-hand: Mean reaction times across blocks for responding to single cues, two-stimuli compounds, and three-stimuli compounds during the negative patterning problem.

The left-hand panel of Figure 4 shows the predictions for the single stimuli, two-stimuli compounds, and triple compounds across the nine blocks of the extended negative patterning training. The right-hand panel of Figure 4 shows the mean reaction times across blocks for responding during the extended negative patterning problem.

First, we checked for successful discrimination learning of the extended negative patterning problem with a 2×9 (Contingency×Block) ANOVA. We found a significant main effect of contingency, *F* (1, 35) = 34.08, *p*<.001, and a significant Contingency×Block interaction, *F* (8, 280) = 45.79, *p*<.001. Second, we compared the accuracy of responding to single cues and two-stimuli compounds which had to be mapped onto one response key. For each participant, we calculated the proportion of correct responses separately for the single cues and the two-stimuli compounds for each block. A Stimulus (single cues vs. two-stimuli compounds)×Block (1–9) ANOVA revealed a significant main effect of stimulus, *F*(1, 35) = 12.60, *p*<.001, and a significant Stimulus×Block interaction, *F*(8, 280) = 2.34, p<.02. Figure 4 (left panel) shows that the proportion of correct responses was lower for two-stimuli compounds than for single cues.

We also compared the speed of responding to single cues and two-stimuli compounds in the same way. A Stimulus (single cues vs. two-stimuli compounds)×Block (1–9) ANOVA revealed a significant main effect of stimulus, *F*(1, 35) = 12.53, *p*<.001, showing faster responding to single cues than to two-stimuli compounds. The Stimulus×Block interaction did not reach significance, *F*(8, 280) = 1.16.

To sum up, compared to Experiment 3 (where the participants worked in a self-paced manner) the time pressure in Experiment 4 reversed the rank order of single cues and two-stimuli compounds. Responding to the single cues was more accurate and faster than responding to the two-stimuli compounds, a clear confirmation of the predictions of the configural theory.

The conclusions drawn from the results of Experiments 3 and 4 are based on cross-experimental comparisons and therefore should be treated with caution. In order to strengthen our conclusions, we compared responding to single stimuli and two-stimuli compounds across the six blocks in Experiment 3 with the first six blocks in Experiment 4. An Experiment (3 vs. 4)×Stimulus (single stimuli vs. two-stimuli compounds)×Block (1–6) ANOVA revealed no main effect of stimulus, *F* <1, but a significant Stimulus×Block interaction, *F* (5, 350) = 2.34, *p*<.05. Most importantly, the analyses yielded a significant Experiment×Stimulus interaction, *F* (1, 70) = 13.55, *p*<.001, as well as a significant Experiment×Stimulus×Block interaction, *F* (3, 350) = 2.24, *p*<.05.

### Conclusions

In a series of four experiments with humans, including Pavlovian SCR conditioning, causal learning, and a speeded classification task, we contrasted the predictions of elemental and configural theories of associative learning regarding different discrimination problems. All the results are in support of the idea that the amount of available processing time is crucial for the appropriateness of the predictions of elemental and configural theories. The configural theory of Pearce holds for observations based upon early stimulus processing (FIR, speeded classification), while with more time available (SIR, causal learning) results conform to the elemental theories.

Each of our Pavlovian conditioning experiments yielded diverging results depending on the time after CS onset. The results obtained for the FIR (1–4 s after CS onset) replicated the findings reported by Redhead and Pearce [1] for autoshaping in pigeons: stronger responses to single stimuli than to two-stimuli compounds as it is predicted by Pearce’s configural theory.

However, for the SIR (4–9 s after CS onset), we observed a pattern of results that is inconsistent with Pearce’s configural theory, but rather supports the elemental theories and the extended configural theory. Similar to the results of the eyeblink conditioning study of Kinder and Lachnit [4], we found no differences in the SIR to the two types of reinforced stimuli in the course of differential conditioning. This was true although we considerably enhanced the time resolution compared to Kinder and Lachnit by reducing the numbers of trials per block in the case of reinforced trials in SCR-conditioning.

The results from our causal learning experiment in which participants worked self-paced replicated the findings from Lachnit et al. [5] who used pictures of fruits as stimuli. As only anticipated by elemental theories, we observed higher responding to two-stimuli compounds than to single cues. In our final experiment, however, in which we explicitly limited the amount of time available to categorize the stimuli, we observed the opposite rank order of responding. Consistent with our findings for the FIR in Experiments 1 and 2, responding to single stimuli was stronger than to two-stimuli compounds.

In all the present experiments, we used the same kind of unimodal visual stimuli (colored rectangles) which were also used by Redhead and Pearce and Kinder and Lachnit. Nevertheless, we observed different pattern of results across the experiments, but consistencies with results of studies using other kinds of stimuli (e. g. Lachnit et al. [5]). Hence, the stimuli cannot be responsible for the differences observed within the present study.

In each of the Experiments 3 and 4, the extended negative patterning problem was trained in an interleaved fashion with an extended positive patterning problem, while in Experiments 1 and 2 only one discrimination problem was presented to the participants. Redhead and Pearce [1] and Kinder and Lachnit [4], however, have shown that it does not matter whether or not extended patterning problems are trained solely or interleaved. Our present results support this conclusion as, for example, the results of Experiment 4 converged more with the FIR data of Experiments 1 and 2 than with the results of Experiment 3.

Instead, it might be more useful to consider some of the properties of the different responses measured on our experiments. For instance, causal ratings are a straightforward measure of outcome expectancy, which are not linked with orienting responses. The parameterization of the SIR as a response antedating the US suggests that this is also a pure measure of outcome expectancy. The FIR, however, measured as a response to CS onset, is known to arise from two different processes. On the one hand, it is a conditioned response (CR), on the other hand it is an orienting response [28], [29]. Hence, FIR results might differ from the other measures due to an additional orienting component.

This similarity between FIR and autoshaping might be the reason that we found support for the configural theory in all of the present SCR conditioning experiments with FIR. Thus, instead of elaborating consequences of the unimodal versus multimodal distinction, it might be more useful to take into consideration that configural theory accounts better for data in which orienting and learning are ‘confounded’ than for rather pure indicators of associative strength, although no Pearce-Hall [35] like mechanism is included in the theory.

The CR part, determined by the strength of the CS-US association, is growing in the course of learning, while the orienting part is not directly related to associative strength. Kaye and Pearce [36] proposed that the strength of orienting is inversely related to the predictive accuracy of the CS. At the outset of training, the predictive accuracy is low and hence the orienting response is high. The overall decline of FIR in both of our SCR experiments well fits this suggestion. In case of FIR the decrement in responding due to decreasing orienting responses exceeds the increase in responding due to increasing associative strength.

Another way to think about reasons for the pattern of results observed is the already introduced suggestion that configural processing dominates early in a trial and elemental processing later. Thus, as far as early responses (FIR and speeded classification in our case) are concerned, configural theory, assuming that configurations enter into associations, best predicts what we observed. Depending on the specific demands of the task (e.g., kind of discrimination problem, covariation of certain stimulus dimensions with reinforcement, etc.) the representations change in the course of stimulus processing. In this way, the distinctiveness of the representations can be enhanced (mirrored by an increase in the discrimination parameter *d* as suggested by the extended configural theory) and by this generalization decreases. Or, analytical (elemental) features get represented, which then can be associated with different outcomes. The “late” responses, SIR and causal learning, taken as a measure of outcome expectancy or ‘pure’ conditioning therefore show a response pattern different from the FIR.

There are different possibilities to further look into this kind of reasoning all having in common that the amount of time available to solve the discrimination explicitly is manipulated. Firstly, the ISI in eyeblink conditioning studies might be decreased. The 1200 ms interval used by Kinder and Lachnit [4], for example, gave the participants plenty of time to process the CSs. It might well be hypothesized that with shorter ISIs earlier processing should be measured and the results should conform to the predictions of configural theory even in eyeblink conditioning. Secondly, in causal learning studies either the presentation time of stimuli or the time available to make the predictions could be manipulated within one experiment..

Taken together, all the observations in the present series of experiments (Pavlovian SCR, causal learning, and speeded classification task) converged and clearly strengthen the idea that the scope of associative learning theories is determined by the available amount of processing time. We should take this into consideration and attend more to this crucial factor in future research.

## Supporting Information

Appendix S1 **Parameter selection for simulations of the elemental models (modified unique cue model and Harris’ model) and configural models (Pearce’s configural theory and extended configural theory) for the two discrimination problems A/B/C+, AB/BC/AC+ vs. ABC- and A+, BC+ vs. ABC-.**
(DOCX)
