# Supplementary material for: Indicators of Early and Late Processing Reveal the Importance of Within-Trial-Time for Theories of Associative Learning
Source: PLoS One. 2013 Jun 24;8(6):e66291. doi: 10.1371/journal.pone.0066291 (PMC3691220; doi:10.1371/journal.pone.0066291)
Supplement: Appendix S1 — Parameter selection for simulations of the elemental models (modified unique cue model and Harris’ model) and configural models (Pearce’s configural theory and extended configural theory) for the two discrimination problems A/B/C+, AB/BC/AC+ vs. ABC- and A+, BC+ vs. ABC-. (DOCX) [file pone.0066291.s001.docx]

Appendix S1

Because our questions are of a more qualitative than a detailed quantitative manner, the exact values of the learning rate parameters and the number of trials are not the main focus. Nevertheless, parameters for each of the theories were kept constant across simulations of the two discrimination problems under examination. Furthermore, as far as possible, parameters were kept constant across the different theories. In coding the stimuli for our simulations, as a first alternative we only coded their colors but neglected the number of rectangles. That way, single stimuli became A, B, or C, two-stimuli compounds became AB, AC, and BC, and the triple compound became ABC. As a second alternative, we took into account the number of rectangles. In order to be able to simulate halves and thirds of stimuli we represented each elementary CS by six units (a1 – a6, b1 – b6, c1 – c6).

With one exception, for all models the learning rate parameter for reinforcement and non-reinforcement (β) was 0.05 (in the case of the Harris model: 0.005). The learning rate parameter for each nominal CS (α) was 1 in Pearce’s configural theory and the extended configural theory. In accordance with Kinder and Lachnit [4], the discrimination parameter *d* was set to 2 for Pearce’s configural theory and to 10 for the extended configural theory. In case of the modified unique cue model we assumed one unique cue for each two-stimuli compound and four unique cues for the three-stimuli compound (one for each of the three binary combinations plus one for the triple combination). The predictions were computed for the salience of the physically presented elements (α = 0.5) being about the same as the salience of the unique cues or the salience of the unique cues being considerably higher (α *_U_* = 0.8). In case of the second alternative we chose a salience of 0.2 for each of the six units (with higher salience the simulations became oscillatory). Unique cues, on the other hand, were represented by single units with a salience of x. The question was how to choose x such that the unique cue is equally or higher salient than an elementary CS comprising six units. To answer this question, the unique cue salience for the equal case was determined first and then the higher salience was derived from this value. More precisely, the salience for the equal case was determined by equating the salience of an elementary CS given as 6 * 0.2^2^ / 6 * 0.2 with the salience of the single unique cue unit given as x^2^ / 6 * 0.2. Solving this equation identifies x = sqrt(0.24) = 0.4899 as the equal salience value. Based on this value we set x = 0.8 for the higher case.

In the case of the Harris model we followed the suggestions by Harris [2] and used 20 elements per CS, an attention buffer gain of 2, and an interconnectivity of 0.5. With respect to β* *we had to depart from the suggestion of Harris: instead of β* *= 0.01 we used β* *= 0.005 because the higher learning rate sometimes resulted in solutions that built up. In addition, we varied the fraction of common elements (0, .1, .5, .9). To simulate the Harris model with halves and thirds of stimuli present we proceeded as follows: First, in case of no common elements for an elementary CS, we split the units representing each CS in halves (for two-stimuli compounds) or thirds (for triple compounds). On each trial, for each compound one half or two thirds of each stimulus' units were randomly selected. Those selected units' activation was then set to zero. Second, in case of common elements for elementary CS, one half or two thirds of the units representing each elementary CS were randomly selected and set to zero on each trial.
